# Engineering the catalytic properties of CeO_2_ catalyst in HCl-assisted propane dehydrogenation by effective doping: A first-principles-based microkinetic simulation

**DOI:** 10.3389/fchem.2023.1133865

**Published:** 2023-03-10

**Authors:** Faheem Jan, Min Yang, Nuodan Zhou, XiaoYing Sun, Bo Li

**Affiliations:** ^1^ Shenyang National Laboratory for Materials Science, Institute of Metal Research, Chinese Academy of Sciences, Shenyang, Liaoning, China; ^2^ School of Materials Science and Engineering, University of Science and Technology of China, Shenyang, Liaoning, China; ^3^ Institute of Catalysis for Energy and Environment, College of Chemistry and Chemical Engineering, Shenyang Normal University, Shenyang, China

**Keywords:** propane dehydrogenation, microkinetic simulation, density functional theory, reaction mechanism, hydrogen chloride

## Abstract

HCl-assisted propane dehydrogenation (PDH) is an attractive route for propene production with good selectivity. In this study, the doping of CeO_2_ with different transition metals, including V, Mn, Fe, Co, Ni, Pd, Pt, and Cu, in the presence of HCl was investigated for PDH. The dopants have a pronounced effect on the electronic structure of pristine ceria that significantly alters the catalytic capabilities. The calculations indicate the spontaneous dissociation of HCl on all surfaces with a facile abstraction of the first hydrogen atom except on V- and Mn-doped surfaces. The lowest energy barrier of 0.50 and 0.51eV was found for Pd- and Ni-doped CeO_2_ surfaces. The surface oxygen is responsible for hydrogen abstraction, and its activity is described by the p-band center. Microkinetics simulation is performed on all doped surfaces. The increase in the turnover frequency (TOF) is directly linked with the partial pressure of propane. The adsorption energy of reactants aligned with the observed performance. The reaction follows first-order kinetics to C_3_H_8_. Furthermore, on all surfaces, the formation of C_3_H_7_ is found as the rate-determining step confirmed by the degree of rate control (DRC) analysis. This study provides a decisive description of catalyst modification for HCl-assisted PDH.

## 1 Introduction

Propylene is a building block of chemical industries used to produce many chemicals such as polypropylene, propylene oxide, acrylonitrile, and acrylic acid*.* Propylene is conventionally obtained by steam and fluid catalytic cracking of naphtha and light diesel (Sattler et al., 2014). However, these methods are thermodynamically limited, give low yield, and have complex separation of the desired products (Che-Galicia et al., 2014). Hence, searching for an alternative method is an urgent task. The large reservoir of shale gas worldwide opens a new channel to provide abundant light alkanes such as propane (Abedin et al., 2020). Under this circumstance, propane direct dehydrogenation (PDH) becomes an alternative and profitable catalytic method of producing propene.

There are two major routes of PDH: non-oxidative and oxidative dehydrogenation. The difference between these two routes is whether an oxidant, such as oxygen, is employed in the reaction*.* The non-oxidative route suffers from two inherent drawbacks, i) the endothermic nature makes it economically unfavorable in that it demands high energy input, and ii) the fast deactivation of the catalyst due to coke formation (Larsson et al., 1996; Lian et al., 2021). The use of oxidants in PDH overcomes the thermodynamics restraint and lowers the reaction temperature (Atanga et al., 2018). More importantly, coke formation is inhibited under oxidizing conditions (Carrero et al., 2014). However, the strong oxidizing ability of oxygen molecules induces the deep reaction of propene, which consequently reduces selectivity. The overoxidation issue is significantly lessened by using “soft oxidants,” including CO_2_, N_2_O, sulfur, and halogens, in propane dehydrogenation (Jiang et al., 2021; Zhi et al., 2022). Compared to an oxygen molecule, soft oxidants possess a much-reduced oxidizing ability that is detrimental to side reactions.

Among the soft oxidants, halogens and their halides are the most promising oxidants for propane dehydrogenation. Halogens (X) can assist the PDH pathways i) by molecular halogens (X_2_), ii) oxyhalogenation (X_2_, O_2_), iii) oxyhalogenation *via* halides (HX, O_2_), and iv) oxyhalogenation *via* molten salts of metal halides (LiX, O_2_). Halogens as an oxidant not only suppress the reactivity toward side reactions but also give a new active site for C-H bond activation. Therefore, the use of halogens over metal oxides and phosphate surfaces (CeO_2_, EuOX, CrPO_4_, and FePO_4_) shows a remarkable propene selectivity of up to 95% with 70% propane conversion at 500 °C compared to conventional catalysts (Xie et al., 2018; Zichittella et al., 2019a; Zichittella et al., 2020a).

CeO_2_ is extensively studied due to its activity toward HX oxidation, alkane, and alkene oxyhalogenation (Scharfe et al., 2020). Xie et al. reported the different transitions and rare metal oxides for PDH in the presence of chlorine. The study shows the instability of Fe_2_O_3_, CuO, and NiO, whereas the high reactivity of oxygen leads to CO_x_ as a major product on the RuO_2_ surface. Among them, a high propylene selectivity of 55% with 38% propane conversion was observed on the CeO_2_ surface in the presence of a halogen (Xie et al., 2018). The high conversion of oxygen is also found on the ceria surface, which leads to increased CO_2_ selectivity of up to 20%. The formation of CO_2_ can be reduced by the addition of dopants to the ceria surface. The nickel-doped ceria decreased the selectivity of CO_2_ to 15%. Furthermore, the nickel-doped ceria shows the highest selectivity of propylene at 80%, while the propane conversion reached 69%. In comparison, the undoped CeO_2_ gives only 55% selectivity with 38% propane conversion (Xie et al., 2018). The surface modification of CeO_2_ with doped metals makes a significant change in the catalytic performance (Su et al., 2018; Yang et al., 2018). However, an understanding of this enhancement induced by dopants is still absent, even though doped ceria has attracted much attention in oxidative dehydrogenation (Yang et al., 2018). Ceria doped with transition metals is an attractive approach that gives a balanced activity and selectivity for propane dehydrogenation. Our previous work identified the role of HBr in PDH for pristine CeO_2_ catalysts. This study revealed that the presence of HBr facilitated the C-H bond activation and altered the conventional reaction pathway. The apparent activation barrier is reduced with the presence of HBr (Jan et al., 2022).

In the current work, we further deepen the understanding of HCl-assisted propane dehydrogenation on metal-doped ceria. The dopant candidates include V, Mn, Fe, Co, Ni, Pd, Pt, and Cu. The geometry and electronic properties of doped ceria are carefully examined and compared. The electronic structure analysis indicated the pronounced effects induced by dopants and their implications on reactivities. The adsorption of reactants and reaction pathways are carefully examined. The thermodynamic and kinetic parameters from DFT calculations are streamed into the microkinetic simulation, and the most promising dopant candidates are proposed and verified. The current work provides a practical optimization method to further improve performance and reveal the origin of enhancement from the doping strategy.

## 2 Computational details

The reported work was performed using the spin-polarized density functional theory (DFT) method with the on-site Coulomb interaction (DFT + U) using the Vienna *ab initio* simulation package (VASP) (Kresse and Hafner, 1993; Kresse and Furthmuller, 1996). The DFT calculations were performed on the most stable CeO_2_ (111) surface according to the literature (Wang et al., 2013; Wang et al., 2015). A plane-wave basis set was used for valance electrons with a cutoff energy of 400 eV. The projector augmented-wave (PAW) method was used to describe ionic cores (Blöchl, 1994; Kresse and Joubert, 1999). The revised Perdew–Burke–Ernzerhof (RPBE) function was used as an exchange-correlation functional (Hammer et al., 1999). The Brillouin-zone integration was performed at a 1 × 2 × one Monkhorst–Pack *k*-point grid for transition-metal-modified M-CeO_2_ (111) surfaces (M = V, Mn, Fe, Co, Ni, Pd, Pt, and Cu). The van der Waals correction was performed by the DFT-D3 method (Grimme et al., 2010). For the description of cerium 4f, localized electrons were treated according to Dudarev et al. (1998). The value of U for this calculation was selected as 5.5 eV, as previously determined (Jan et al., 2022). The calculation was converged for all optimized structures to an accuracy of 1 × 10^−5^ eV, and force tolerance is 0.03 eV/Å.

**FIGURE 1:**
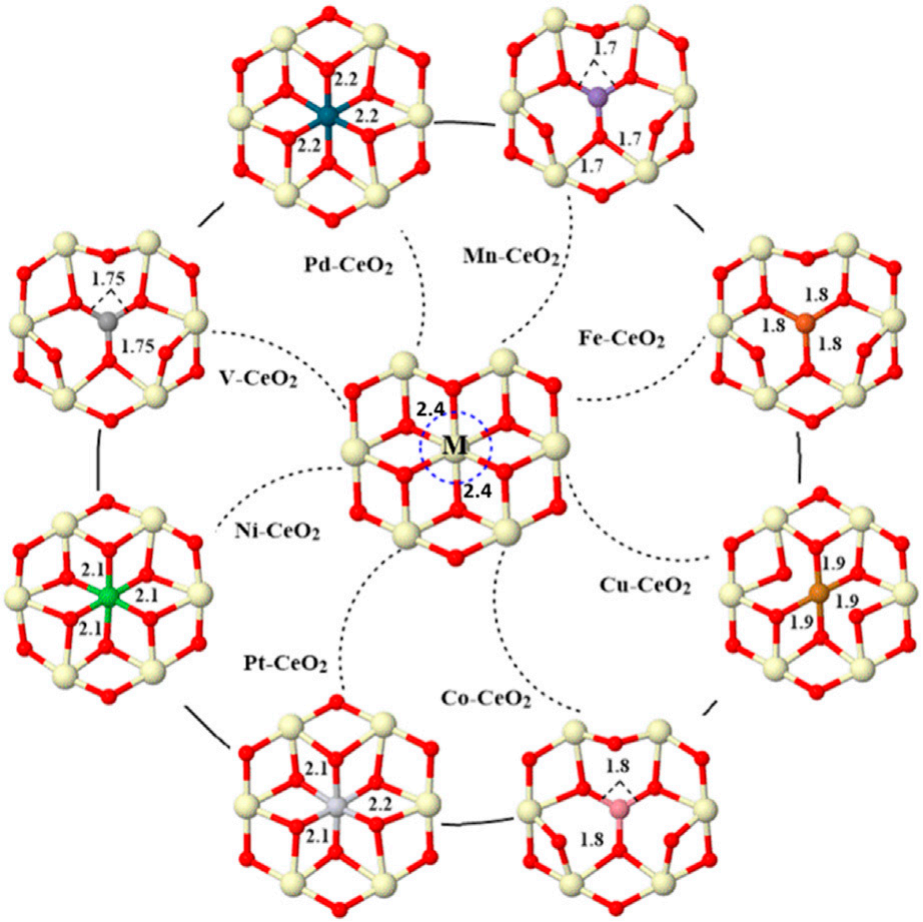
Optimized geometries of all doped surfaces. One of the surface cerium atoms is replaced with a dopant metal (Pd, Mn, Fe, Cu, Co, Pt, Ni, and V). The bond distance between the dopant and the first neighbor oxygen is indicated in Å. Cerium is light yellow, and oxygen is red.

A nine-atomic-layer slab with a 4 × 3 supercell in which the bottom six layers are kept fixed is used to model the ceria surface. The thickness of the vacuum was taken as 15 Å in the *z* direction. One cerium atom is substituted by transition metal M (M = V, Mn, Fe, Co, Ni, Pd, Pt, and Cu), as shown in Figure 1. The adsorption energy E_ads_ was calculated as

$$E_{ads}=\left[E_{adsorbate/slab}-\left(E_{slab}+E_{adsorbate}\right)\right],$$ (1)

in which *E*_*adsorbate/slab*_ represents the total energy of interaction species with the slab, *E*_*slab*_ is the individual energy of the CeO_2_/M-CeO_2_ surface, and the adsorbate represents the isolated molecules of C_3_H_8_, O_2_, and HCl, respectively. The climbing image nudged elastic band (CINEB) method was used to compute the reaction path (Henkelman et al., 2000). The true transition state was verified by frequency calculation on each surface.

The bonding strength is analyzed with crystal orbital Hamilton population (COHP) analysis (Dronskowski and Bloechl, 1993). The microkinetics analysis was performed by the MKMCXX simulation package (Filot et al., 2014). It is noted that microkinetic simulation is a mean-field method that discarded surface heterogeneity and dynamics evolution. Although there are some deficiencies, it is still a cost-effective way to mimic surface catalytic reactions. The Gibbs free energy with zero-point energy correction (ZPE) was calculated from DFT frequency calculations at 773 K. The ideal gas approximation was used for the entropy calculation of gas-phase species, while harmonic approximation was used for surface species. The reaction rates for adsorption/desorption species were calculated using Hertz–Knudsen kinetics. The reaction rate for elementary reactions is given by a set of ordinary differential equations as

$$r_{j}=k_{j}\prod_{i}c_{i},$$ (2)

where *r*_*j*_ is the rate of the elementary reaction *j*, *k*_*j*_ is the rate constant of *j*, and *c*_*i*_ represents the concentration of species *i* in a given reaction. The rate constant for the adsorption step is calculated as

$$k_{ads}=\frac{PA}{\sqrt{2\pi mk_{b}T}}.$$ (3)

In the equation, *P* denotes the partial pressure of the gaseous molecule, *A* is the surface area on which the adsorbed molecule is present, *m* denotes the mass of the reactant, and *T* and *k*_*b*_ are the temperature and Boltzmann’s constant, respectively. However, for the surface reaction, the rate constant of the forward/backward reactions is calculated as

$$k_{r}=\frac{Q^{\neq }}{Q_{X}}\;\frac{k_{b}T}{h}\;\exp\left(-\frac{\Delta E^{\neq }}{k_{b}T}\right),$$ (4)

where *X* represents the reacting species, *≠* shows the transition state, *k*_*r*_ denotes the reaction rate for the forward/backward reaction, and *Q* and *h* represent the partition function and Planck’s constant, respectively.

## 3 Results and discussion

### 3.1 Structure and properties of doped ceria

The distance between the dopant and the first neighbor oxygen atom is shortened after doping, as shown in Figure 1. For pristine CeO_2_, the bond distance between Ce and O is 2.4 Å, while the distance between the metal dopant and oxygen is within a range of 1.7–2.2 Å after metal doping. Note also that the coordination number of the dopant is varied due to different valences. For pristine CeO_2_(111), Ce in the top layer has bonded with six oxygen atoms; three reside in the top layer, and the others are in the subsurface. Fe, Cu, Co, V, and Mn have less coordination, while Pt, Ni, and Pd have the same bonding oxygen atoms with a pristine surface. The introduction of dopants effectively modified the electronic structure as revealed from charge, PDOS, and COHP analysis. First, all dopant metals give electrons and become positively charged, as shown in Table 1.

**TABLE 1:** Bader charge analysis of CeO_2_ and doped CeO_2_ surfaces. The * sign indicates the charge on the Ce atom in pristine CeO_2_.

Surface	Charge	
	M |*e*|	O |*e*|
CeO_2_	2.40*	−1.17
Mn–CeO_2_	3.15	−1.09
V–CeO_2_	1.89	−0.98
Fe–CeO_2_	3.39	−1.14
Co–CeO_2_	2.83	−1.05
Ni–CeO_2_	1.71	−0.98
Cu–CeO_2_	2.04	−1.13
Pd–CeO_2_	1.25	−0.96
Pt–CeO_2_	1.35	−0.97

**FIGURE 2:**
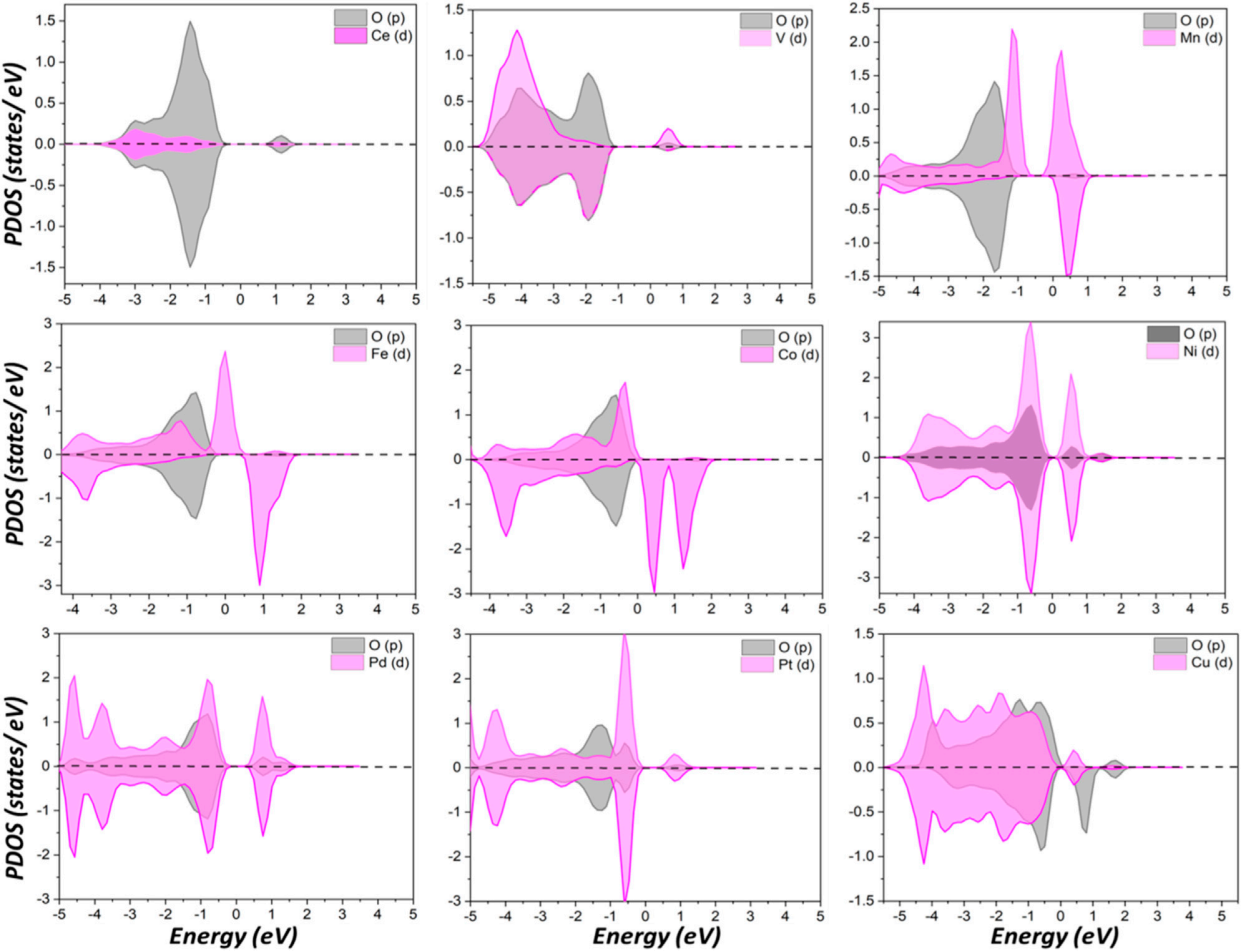
Partial density of states (PDOS) of surface oxygen p orbitals and doped metal d orbitals.

Compared to Ce, Mn, Fe, and Co donated more electrons to surrounding atoms and became more positively charged, while the others have fewer transferred charges than Ce. PDOS analysis of surface oxygen shown in Figure 2 clearly indicated pronounced effects on the electronic structure caused by dopants. New states appeared within the gap between the top of the valence band and the bottom of the conduction band after metal doping that are not present for pristine CeO_2_. This observation is valid for all metal dopants except V and Mn. For the latter two, the conduction band is shifted toward the Fermi level. As surface oxygen will abstract hydrogen from propane, as discussed in the following, a doped surface will have a different behavior for C-H bond activation compared to pristine ceria, as PDOS suggests.

**FIGURE 3:**
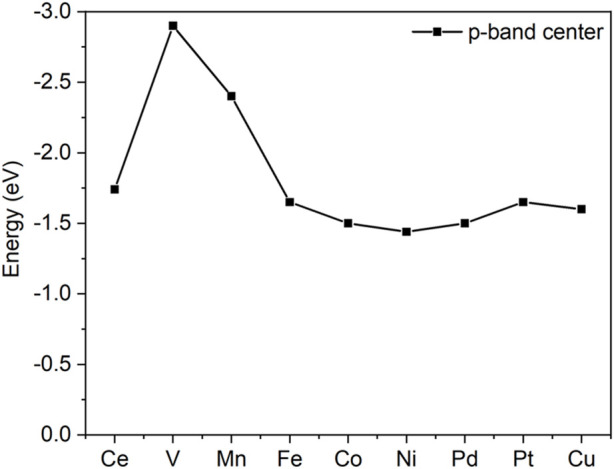
p-band centers of the surface oxygen of doped and pristine ceria.

The activities of oxygen are also clearly indicated from the O p-band center analysis, as shown in Figure 3. Similar to the famous d-band center notation, the p-band center recently exhibited an ability to describe the activity of oxygen (Liu et al., 2019). As shown in Figure 3, the p-band centers of V and Mn are far from the Fermi level, which implies their inferior reactivities compared with others.

### 3.2 HCl and propane adsorption

HCl is a polar molecule that is more active than a propane molecule. First, the adsorption of HCl is explored on doped and pristine CeO_2_. The structure optimization indicated that HCl has a spontaneous dissociation upon approaching the surface, as shown in Supplementary Figure S1. The dissociated Cl and H bind at metal and oxygen sites, respectively. HCl dissociation is a highly exothermic process, as indicated in Supplementary Table S1, and all doped surfaces have a stronger tendency for dissociation than pristine CeO_2,_ which underscored the doping effects. The Ni-doped CeO_2_ has the most exothermic dissociation of −2.5 eV. The bond distance between Cl and the metal dopant is in the range of 2.2–2.76 Å, which falls into the sum of covalent radius. The strong bonding is also evident from the COHP analysis, as shown in Supplementary Figure S2, as the value of ICOHP is quite large. The addition of Cl also further engineered the electronic structure of the doped CeO_2_ surface due to its strong withdrawing electron ability. All metal dopants donated electrons to adsorbed Cl and became more positively charged. Hence, HCl dissociation induced a significant charge re-distribution that created more polar surface sites. The other fragment H* species became a surface hydroxyl (OH*) with a bond distance of 0.99 Å.

**FIGURE 4:**
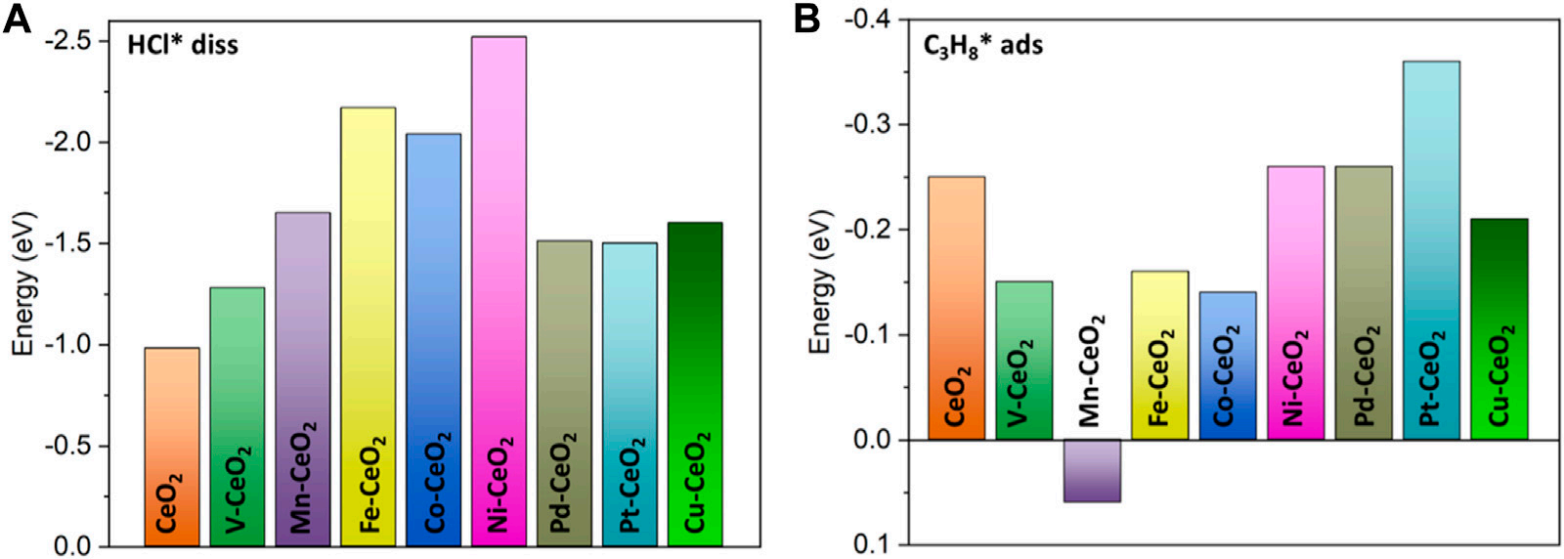
**(A)** Dissociation energy of the HCl molecule; **(B)** adsorption energy of the propane molecule.

The adsorption of propane is also investigated, as shown in Figure 4B. As expected, the propane adsorption belongs to weak physisorption with adsorption energy in a range of −0.14 to −0.36 eV, except for Mn doping, which has a slight endothermic adsorption of 0.06 eV. Pt-doped ceria has the highest adsorption energy at −0.36 eV, and the distance between the adsorbed propane and the surface is in the range of 2.8–3.3 Å.

### 3.3 C-H bond activation

**FIGURE 5:**
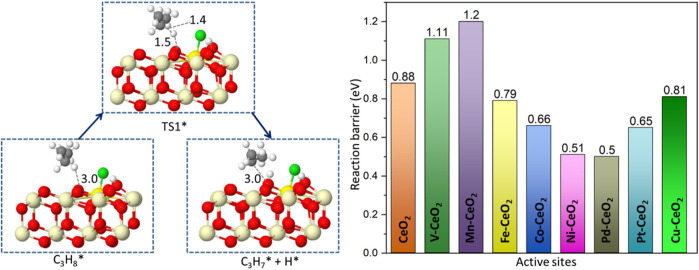
Reaction path and reaction barrier for the abstraction of a first hydrogen atom with a Gibbs free energy barrier (TS1) of all catalytic surfaces at 773 K. The yellow sphere represents the dopant position.

The C-H bond activation is the core issue in propane dehydrogenation as two hydrogen abstractions are required to reach the desired product, propene. Due to its strong bonding energy, C-H activation in propane often is deemed as the descriptor for reactivity in dehydrogenation. On the chlorinated surface, the adsorbed propane proceeded *via* the first hydrogen abstraction from secondary carbon and formed the isopropyl species, as shown in Figure 5. The surface oxygen acts as an active site to attack the C-H bond. At the transition state, the C-H bond is stretched to be around 1.4 Å from its original bond distance of 1.1 Å. Pd-, Ni-, Pt-, and Co-doped ceria demonstrated relatively low barriers of 0.50, 0.51, 0.65, and 0.66 eV, respectively. In contrast, the energy barrier on the pristine CeO_2_ surface is calculated to be 0.88 eV. In contrast, V- and Mn-doped ceria possessed higher energy barriers of 1.11 and 1.20 eV that are larger than pristine CeO_2_. This observation is consistent with the previous oxygen p-band center analysis shown in Figure R5. The best dopants, Ni and Pd, have been suggested to have superior performance in propane dehydrogenation from experimental reports (Su et al., 2018; Xie et al., 2018).

After the C-H bond was broken, the dissociated hydrogen moved toward the surface oxygen and formed surface hydroxyl. The other fragment, C_3_H_7_, is attracted by surface Cl* due to its electron affinity to form C_3_H_7_Cl*. This is an exothermic process, and the energy is decreased by over 1 eV for all doped ceria surfaces. C_3_H_7_Cl* is an important intermediate in the dehydrogenation process and opens a new pathway for propylene formation (Ding et al., 2013; Xie et al., 2018; Zichittella et al., 2020b). In contrast, the direct conversion of propane to propene without the involvement of C_3_H_7_Cl* is generally considered negligible in the oxyhalogenation process, which clearly indicates the importance of C_3_H_7_Cl*. (Zichittella et al., 2017; Xie et al., 2018; Zichittella et al., 2018; Zichittella et al., 2019b). The newly formed C_3_H_7_Cl* is located above the surface at a distance in a range of 2.5–3.3 Å, as shown in Supplementary Figure S4.

### 3.4 Propene formation

**FIGURE 6:**
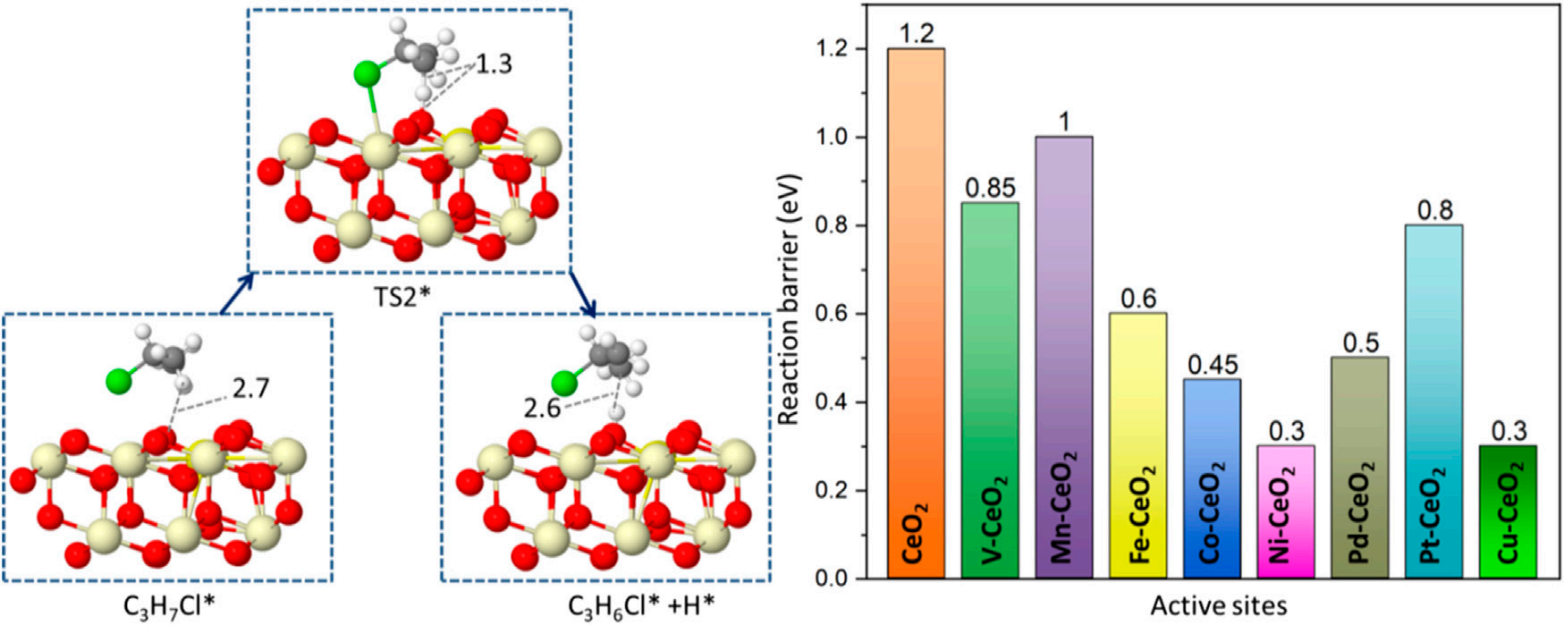
Reaction path and reaction barrier for the removal of the second H atom from C_3_H_7_Cl* with the Gibbs free energy barriers (TS2) of all catalytic surfaces.

**SCHEME 1:**
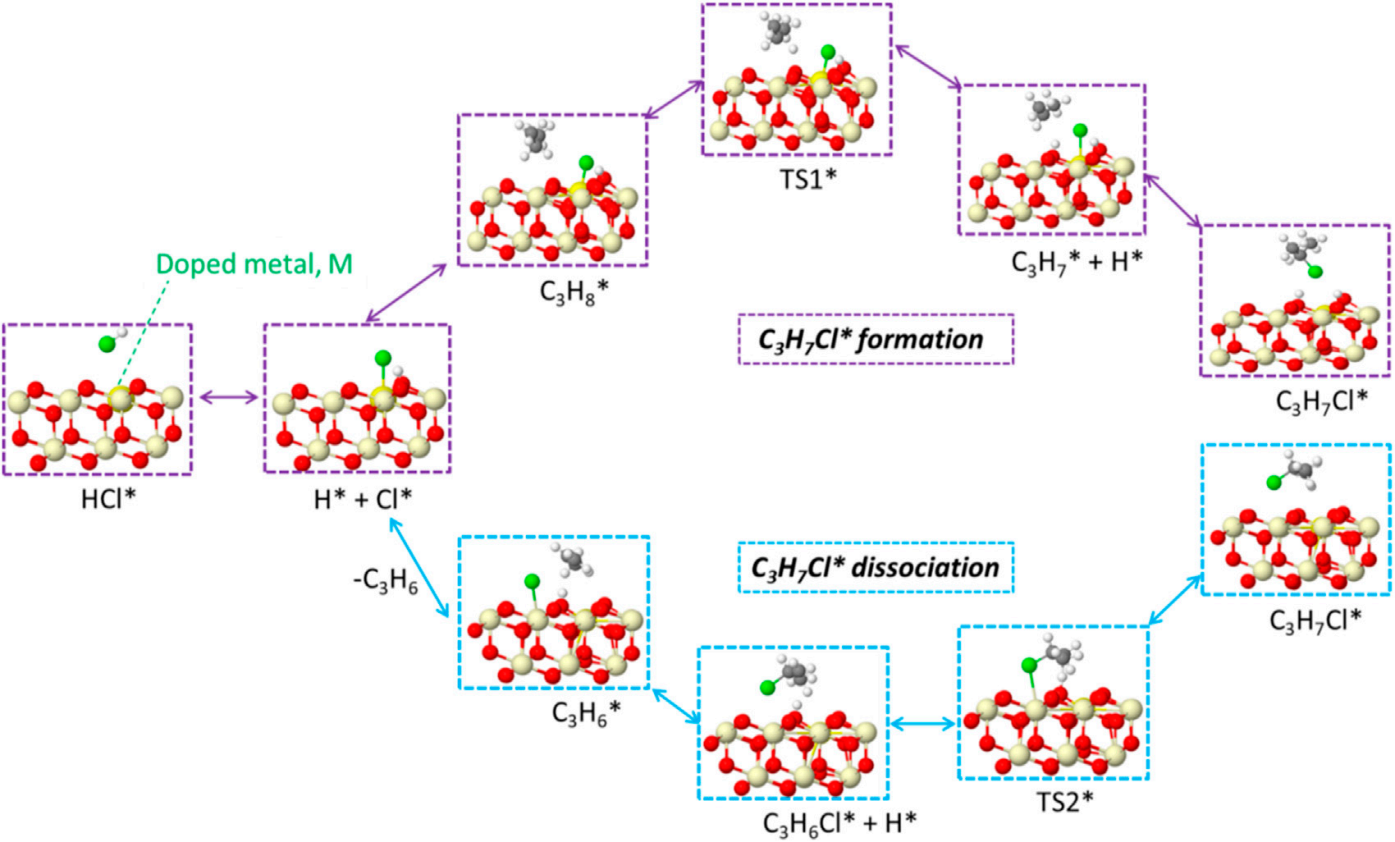
Schematic representation of the elementary steps for PDH.

The generated C_3_H_7_Cl* will further undergo dehydrogenation, as shown in Figure 6. The C_3_H_7_Cl* dissociation leads to the formation of propylene, as shown in Figure 6. A two-step dissociation is calculated on all surfaces. The first step starts with the removal of a hydrogen atom from the primary carbon and leads to the formation of a C_3_H_6_Cl* species. This elementary step proceeds with an energy barrier, as indicated in Figure 6. Among examined doped ceria, Ni, Cu, Pd, and Co have the lowest barriers that are no more than 0.5 eV. It is noted that the barrier associated with the second hydrogen abstraction is smaller than that of the first hydrogen abstraction, as shown in Figure 6. It was also found that Ni-doped ceria possessed the lowest barriers for two successive hydrogen abstractions among investigated dopants. Compared to pristine CeO_2_, all doped catalysts decreased the second hydrogen abstraction barrier and showed a better catalytic ability. In the next step, C_3_H_6_Cl* species proceed *via* a spontaneous dissociation with no barrier, and the detached Cl* rebounded with the surface. This step is exothermic for all investigated doped ceria, as indicated in Supplementary Table S1. A catalytic cycle is completed after the targeted product C_3_H_6_ release, as shown in Scheme 1. Overall, the key intermediate, C_3_H_7_Cl, was a bridge between C-H bond activation in propane and propene formation, as illustrated in Scheme 1. The doped ceria shows a remarkable reduction of hydrogen abstraction barrier compared with the pristine sample.

The surface oxygen sites are covered with H-atoms after dehydrogenation. The interactions between surface oxygen and hydrogen lead to water formation, as shown in Supplementary Figure S7, with a low barrier of 0.1 eV. After water release, an oxygen vacancy site is formed that will be subsequently filled by oxygen molecule dissociation.

## 4 Microkinetics simulations

Microkinetics simulation was performed based on DFT calculations to determine the influence of different reaction conditions on the reaction kinetics and evaluate the most favorable catalytic surface. The main elementary steps are divided into adsorption, desorption, and surface reactions, as listed in Supplementary Table S2. From DFT calculations, the Gibbs free energy, rate constant, and reaction order are calculated at 773 K and 1 bar. For all elementary steps, the rate constants with pre-factors are listed in Supplementary Table S2 of the supporting information file.

**FIGURE 7:**
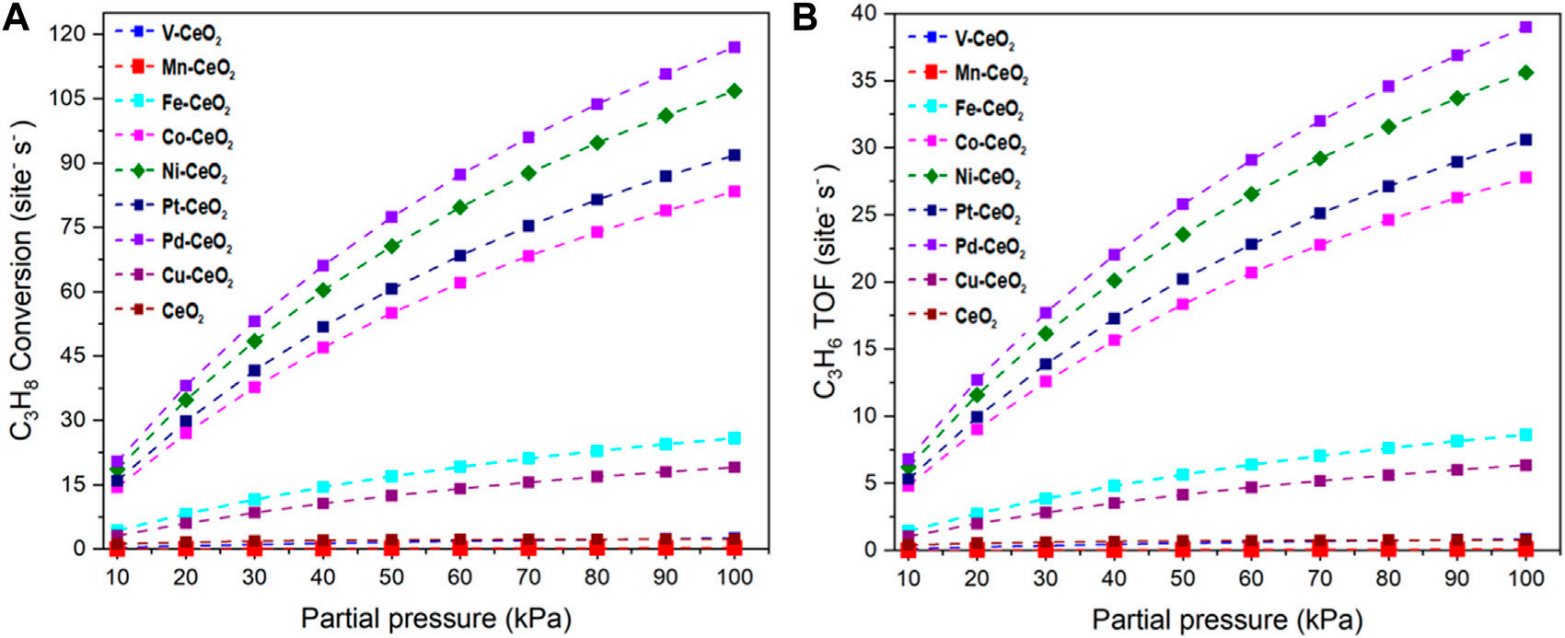
**(A)** C_3_H_8_* conversion rate. **(B)** C_3_H_6_ formation TOF under different partial pressures of propane.

The relationship between the reaction rates with the partial pressure of the reactant is shown in Figure 7. The investigated catalysts exhibited different dependencies on reactant pressure. Pd-, Ni-, Pt-, and Co-doped ceria have a strong positive correlation of TOF with pressure. TOF has a sharp increase with the pressure increase, while the other catalysts have a rather mild TOF increase.

**FIGURE 8:**
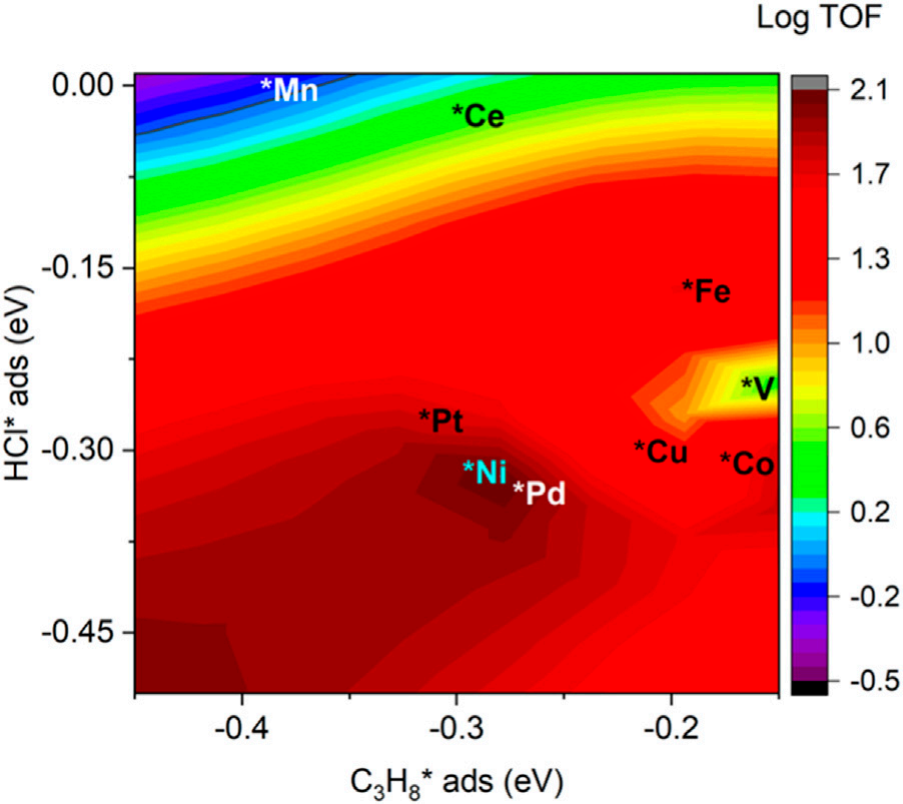
Relationship between TOF (C_3_H_8_ conversion) and the adsorption energies of HCl and C_3_H_8_.

The adsorption of propane and HCl regulates the performance of investigated catalysts, as shown in Figure 8. From the heat map, the modest binding energies of propane and HCl yield the best performance, which is around −0.3 eV for both cases. It is noted that Ni, Pt, and Pd possessed a bigger TOF than the other doped and pristine CeO_2_ samples. This is consistent with previous electronic structure and pathway analysis. DRC analysis was performed to identify the critical step among the elementary steps. The DRC coefficient for C_3_H_7_* formation was found to be unity for all investigated catalysts, as shown in Supplementary Table S3.

Therefore, it is verified that the formation of C_3_H_7_* is the rate-determining step. C_3_H_7_* is formed by the abstraction of the first hydrogen from a secondary carbon atom. Further analysis indicates that the reaction follows the first-order kinetics to C_3_H_8_, which further confirms that the reaction kinetics are directly linked with the propane concentration.

## 5 Conclusion

In conclusion, the modification of ceria with metal dopants for PDH in the presence of HCl was studied with DFT-based microkinetics simulation. The charge analysis clearly indicated that dopants caused the charge transfer and created local polarization that increased the reactivities accordingly. The surface oxygen is identified as the active site to abstract hydrogen, and its activities are tuned by different dopants. The reaction starts with the spontaneous dissociation of the HCl molecule. The propyl species spontaneously attacks the adsorbed Cl* and gives the intermediate C_3_H_7_Cl* that, upon dissociation, results in the formation of propene. The catalytic performance of Ni- and Pd-CeO_2_ is found to be very promising for PDH. The TOF of propane conversion and propene formation is enhanced and directly related to the partial pressure of propane. DRC coefficient is found as unity for the formation of C_3_H_7_*, which confirmed that the first hydrogen removal is a rate-determining step on all catalytic surfaces. The reaction follows the first-order kinetics to C_3_H_8_. Overall, the current study provides unique insight into the reaction mechanism of HCl-assisted propane dehydrogenation and paves the way for future optimization.

## Data Availability

The original contributions presented in the study are included in the article/Supplementary Material; further inquiries can be directed to the corresponding authors.
