# Supplementary material for: Engineering the catalytic properties of CeO2 catalyst in HCl-assisted propane dehydrogenation by effective doping: A first-principles-based microkinetic simulation
Source: Front Chem. 2023 Mar 10;11:1133865. doi: 10.3389/fchem.2023.1133865 (PMC10036589; doi:10.3389/fchem.2023.1133865)
Supplement: Supplementary file 1 [file DataSheet1.pdf]

Engineering the catalytic properties of CeO_2 Catalyst in HCl-assisted propane dehydrogenation by effective doping: a first-principles based microkinetic simulation

Faheem Jan[a,b], Min Yang[a,b], Zhou Nuodan[a,b], XiaoYing Sun[c]* and Bo Li[c]*

[a]Shenyang National Laboratory for Materials Science, Institute of Metal Research, Chinese Academy of Sciences, Shenyang 110016, Liaoning, People's Republic of China

[b]School of Materials Science and Engineering, University of Science and Technology of China, Shenyang 110016, Liaoning, People's Republic of China

[c]Institute of Catalysis for Energy and Environment, College of Chemistry and Chemical Engineering, ShenYang Normal University, ShenYang 110034, China

Corresponding Author

* Bo Li: e-mail, boli@synu.edu.cn; tel, 86-24-83970027; fax, 86-24-83970019.

* XiaoYing Sun: email: sunxiaoying78@163.com

Adsorption & dissociation of HCl

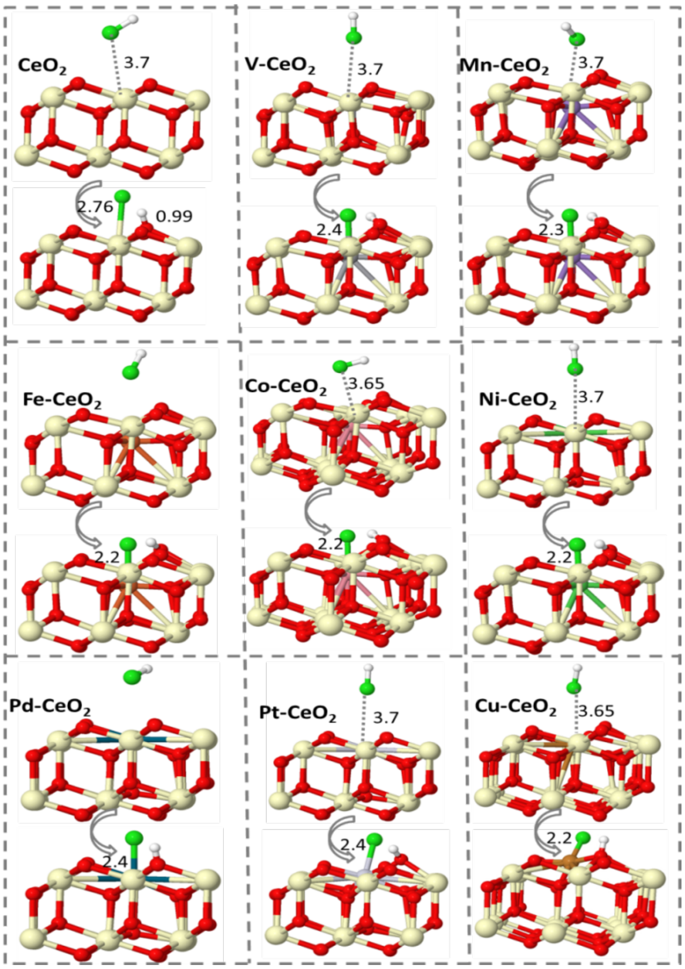

Figure S1. The adsorption and dissociation of HCl molecule on CeO_2 and M-CeO_2 surfaces.

COHP analysis

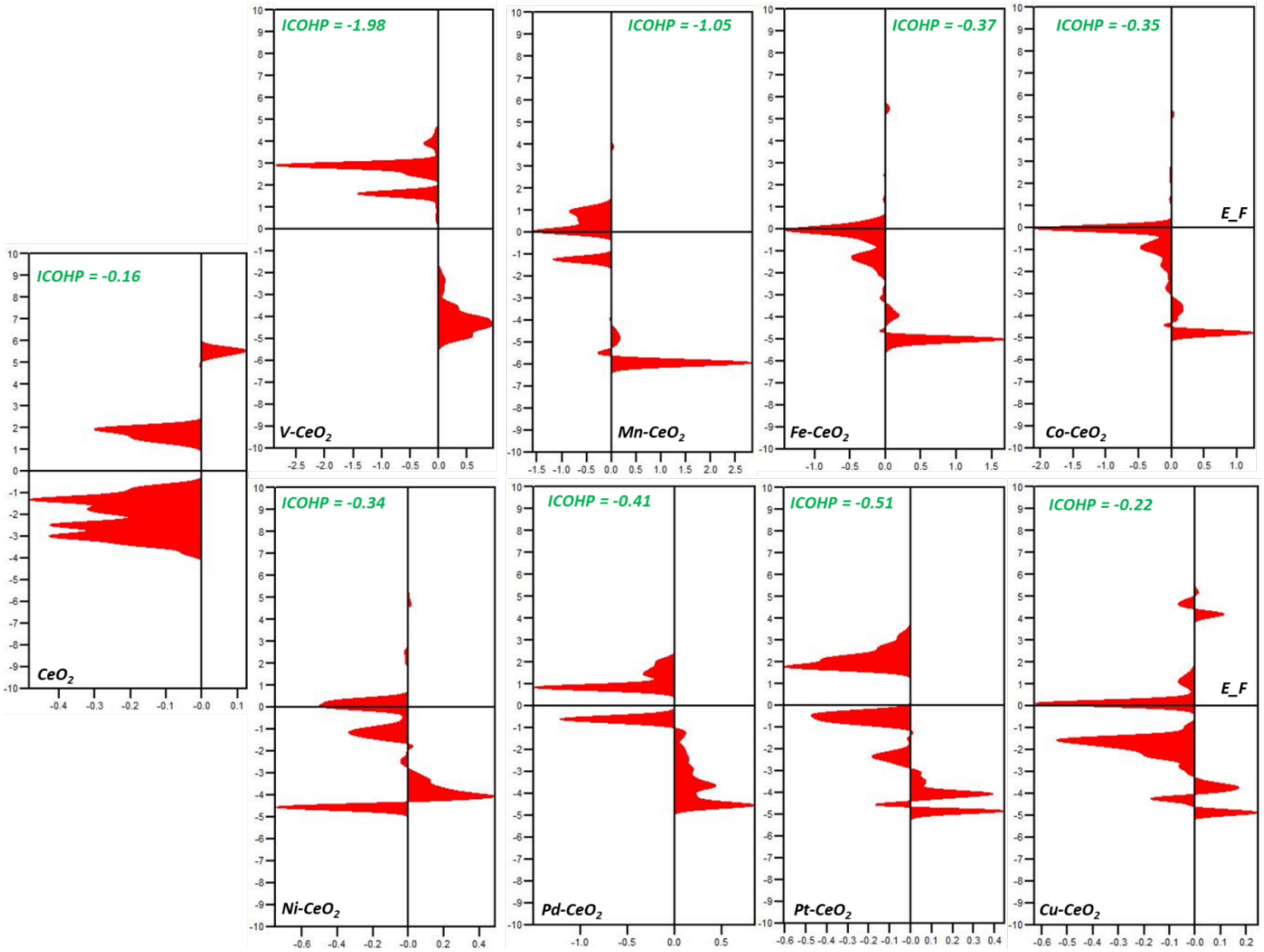

Figure S2. The COHP analysis with ICOHP values inside.

Propane adsorption

The adsorption of propane on all catalytic surfaces as shown in the Figure below,

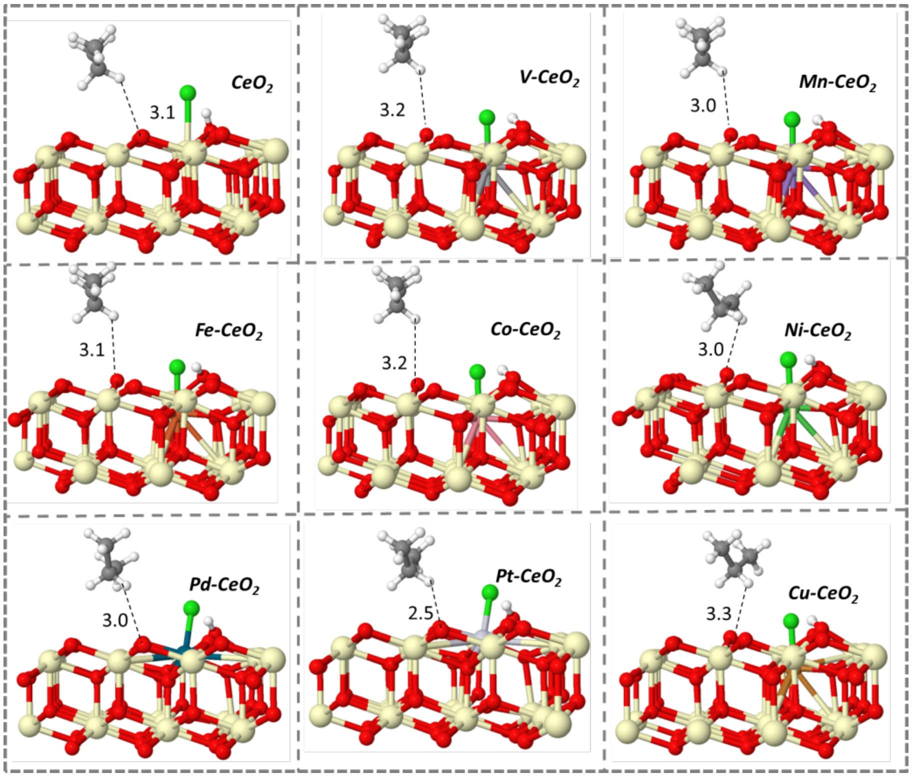

Figure S3. The adsorption of C_3H_8 molecule on all surfaces.

Formation of C_3H_7Cl*

The newly formed C_3H_7Cl* intermediate species.

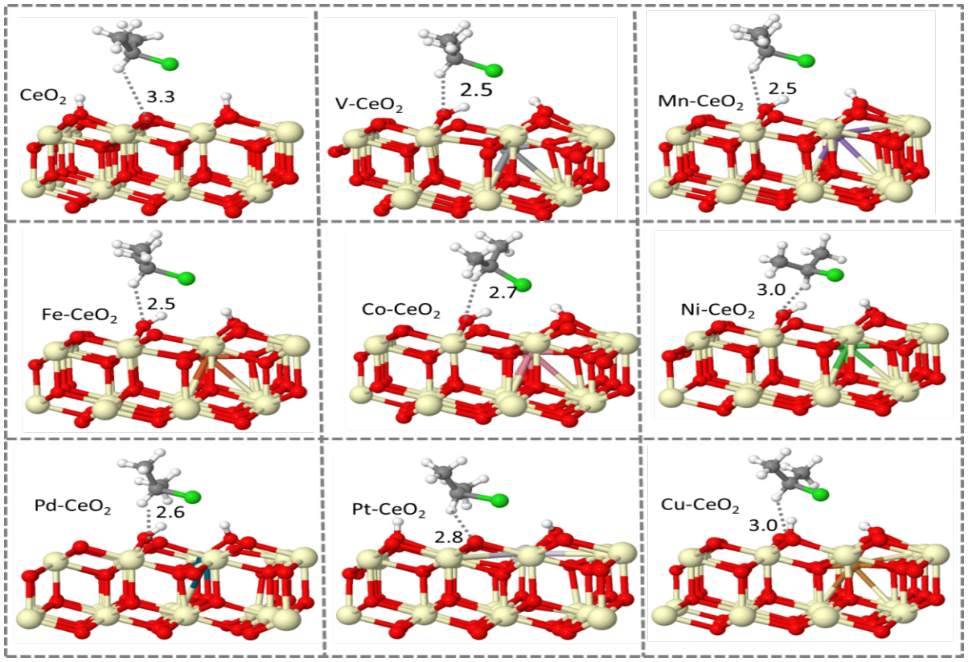

Figure S4. Formation intermediate C_3H_7Cl* species.

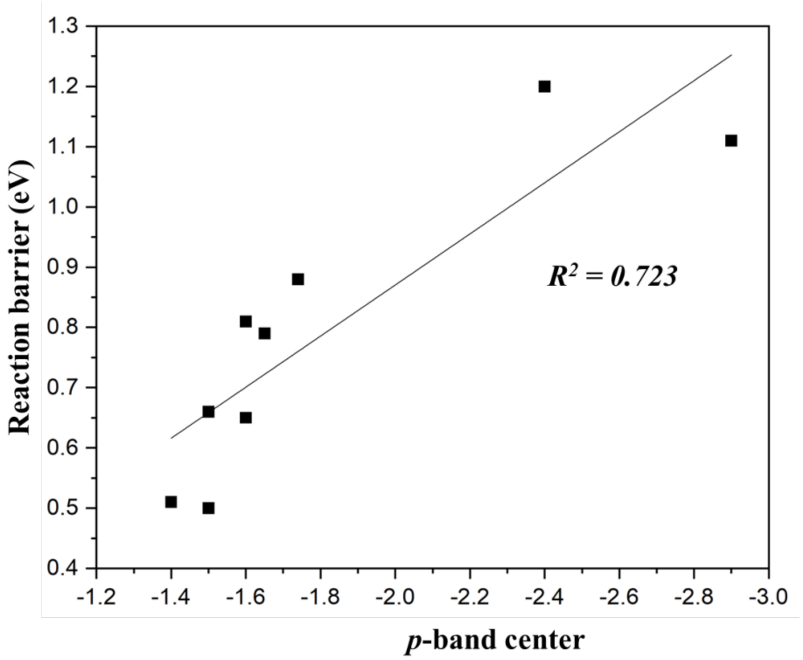

Figure S5. Relation between the calculated reaction barrier and p-band center.

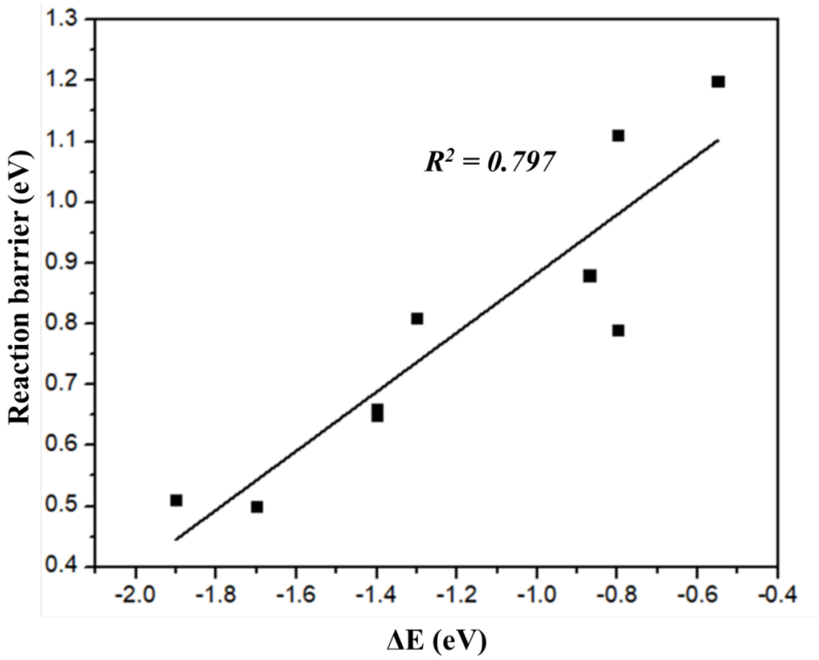

Figure S6. BEP relation for the first C-H bond activation in propane molecule.

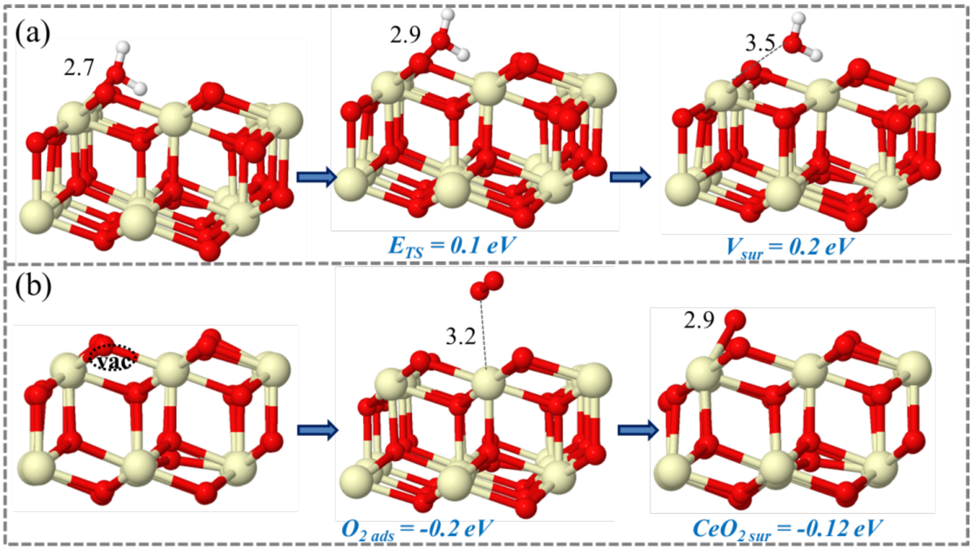

Figure S7. (a) The reaction pathway of water formation (b) the reaction pathway of oxygen vacancy healing

Table S1. Adsorption and dissociation of HCl and C_3H_8 molecules on all surfaces.

Surface	HCl diss	C_3H_8* ads	C_3H_6*
CeO_2	-0.98	-0.25	-0.04
V-CeO_2	-1.28	-0.15	-0.45
Mn-CeO_2	-1.65	0.06	-0.39
Fe-CeO_2	-2.17	-0.16	-0.37
Co-CeO_2	-2.0	-0.14	-0.70
Ni-CeO_2	-2.52	-0.26	-0.32
Pd-CeO_2	-1.51	-0.26	-0.74
Pt-CeO_2	-1.50	-0.36	-0.9
Cu-CeO_2	-1.60	-0.21	-0.86

Elementary steps

The main elementary steps on all catalytic surfaces are given in Table S1 with rate constant and pre-factor at 773 K

Table S2. Shows the elementary steps with the rate constant and pre-factor at 773 K

Reactions	Rate constant		Pre-factor	
*CeO_2 surface	k_{fwd}	k_{bwd}	*fwd*	*bwd*
HCl + * → HCl*	1.04E+08	3.11E+13	1.04E+08	2.41E+18
$CH_3CH_2CH_3$ + * → $CH_3CH_2CH_3$*	1.04E+08	2.88E+13	1.04E+08	2.04E+19
CH_3CHCH_2* → CH_3CHCH_2 + *	1.44E+08	1.54E+18	1.44E+08	7.01E+18
HCl* + * → H* + Cl*	1.67E+13	1.65E+13	1.67E+13	1.67E+13
$CH_3CH_2CH_3$* + *→CH_3CHCH_3* + H*	4.98E+07	2.71E+11	1.67E+13	1.67E+13
CH_3CHCH_3* + Cl*→$CH_3CHClCH_3$* + *	1.67E+13	1.17E+11	1.67E+13	1.67E+13
$CH_3CHClCH_3$* + * → $CH_3CHClCH_2$* + H*	1.89E+04	5.60E+09	1.67E+13	1.67E+13
$CH_3CHClCH_2$* + *→CH_3CHCH_2* + Cl*	1.67E+13	5.32E+12	1.67E+13	1.67E+13
*V-CeO_2				
HCl + * → HCl*	1.04E+08	3.11E+13	1.04E+08	2.41E+18

$CH_3CH_2CH_3$ + * → $CH_3CH_2CH_3$*	1.04E+08	1.11E+14	1.04E+08	2.04E+19
CH_3CHCH_2* → CH_3CHCH_2 + *	1.44E+08	1.54E+18	1.44E+08	7.01E+18
HCl* + * → H* + Cl*	1.67E+13	1.64E+13	1.67E+13	1.67E+13
$CH_3CH_2CH_3$* + *→CH_3CHCH_3* + H*	1.98E+06	2.44E+05	1.67E+13	1.67E+13
CH_3CHCH_3* + Cl*→$CH_3CHClCH_3$* + *	1.67E+13	6.01E+05	1.67E+13	1.67E+13
$CH_3CHClCH_3$* + * → $CH_3CHClCH_2$* + H*	1.41E+08	9.02E+11	1.67E+13	1.67E+13
$CH_3CHClCH_2$* + *→CH_3CHCH_2* + Cl*	1.67E+13	2.41E+10	1.67E+13	1.67E+13
*Mn-CeO_2				
HCl + * → HCl*	1.04E+08	3.11E+13	1.04E+08	2.41E+18
$CH_3CH_2CH_3$ + * → $CH_3CH_2CH_3$*	1.04E+08	1.42E+14	1.04E+08	2.04E+19
CH_3CHCH_2* → CH_3CHCH_2 + *	1.44E+08	1.54E+18	1.44E+08	7.01E+18
HCl* + * → H* + Cl*	1.67E+13	1.64E+13	1.67E+13	1.67E+13
$CH_3CH_2CH_3$* + *→CH_3CHCH_3* + H*	3.34E+05	4.00E-01	1.67E+13	1.67E+13
CH_3CHCH_3* + Cl*→$CH_3CHClCH_3$* + *	1.67E+13	5.64E+00	1.67E+13	1.67E+13
$CH_3CHClCH_3$* + * → $CH_3CHClCH_2$* + H*	2.70E+06	4.53E+10	1.67E+13	1.67E+13
$CH_3CHClCH_2$* + *→CH_3CHCH_2* + Cl*	1.67E+13	3.36E+09	1.67E+13	1.67E+13
*Fe-CeO_2				
HCl + * → HCl*	1.04E+08	3.11E+13	5.11E+07	5.59E+07
$CH_3CH_2CH_3$ + * → $CH_3CH_2CH_3$*	1.04E+08	1.00E+15	5.11E+07	5.11E+07
CH_3CHCH_2* → CH_3CHCH_2 + *	1.44E+08	1.54E+18	8.63E+00	-3.70E-07
HCl* + * → H* + Cl*	1.67E+13	1.64E+13	2.95E+07	3.16E+07
$CH_3CH_2CH_3$* + *→CH_3CHCH_3* + H*	1.71E+08	1.37E+07	8.63E+00	7.78E-08
CH_3CHCH_3* + Cl*→$CH_3CHClCH_3$* + *	1.67E+13	2.32E+06	8.64E+00	9.95E-03
$CH_3CHClCH_3$* + * → $CH_3CHClCH_2$* + H*	2.02E+09	6.95E+07	8.63E+00	4.57E-08
$CH_3CHClCH_2$* + *→CH_3CHCH_2* + Cl*	1.67E+13	8.26E+10	7.44E+01	6.58E+01
*Co-CeO_2				
HCl + * → HCl*	1.04E+08	3.11E+13	1.04E+08	2.41E+18
$CH_3CH_2CH_3$ + * → $CH_3CH_2CH_3$*	1.04E+08	2.32E+15	1.04E+08	2.04E+19
CH_3CHCH_2* → CH_3CHCH_2 + *	1.44E+08	1.54E+18	1.44E+08	7.01E+18
HCl* + * → H* + Cl*	1.67E+13	1.64E+13	1.67E+13	1.67E+13
$CH_3CH_2CH_3$* + *→CH_3CHCH_3* + H*	1.26E+09	4.78E+06	1.67E+13	1.67E+13
CH_3CHCH_3* + Cl*→$CH_3CHClCH_3$* + *	1.67E+13	2.56E+04	1.67E+13	1.67E+13
$CH_3CHClCH_3$* + * → $CH_3CHClCH_2$* + H*	2.48E+10	5.00E+06	1.67E+13	1.67E+13
$CH_3CHClCH_2$* + *→CH_3CHCH_2* + Cl*	1.67E+13	4.24E+06	1.67E+13	1.67E+13
*Ni-CeO_2				
HCl + * → HCl*	1.04E+08	3.11E+13	1.04E+08	2.41E+18
$CH_3CH_2CH_3$ + * → $CH_3CH_2CH_3$*	1.04E+08	1.00E+15	1.04E+08	2.04E+19
CH_3CHCH_2* → CH_3CHCH_2 + *	1.44E+08	1.54E+18	1.44E+08	7.01E+18
HCl* + * → H* + Cl*	1.67E+13	1.64E+13	1.67E+13	1.67E+13
$CH_3CH_2CH_3$* + *→CH_3CHCH_3* + H*	6.97E+08	8.11E+05	1.67E+13	1.67E+13
CH_3CHCH_3* + Cl*→$CH_3CHClCH_3$* + *	1.67E+13	1.46E+00	1.67E+13	1.67E+13
$CH_3CHClCH_3$* + * → $CH_3CHClCH_2$* + H*	5.20E+12	5.63E+10	1.67E+13	1.67E+13
$CH_3CHClCH_2$* + *→CH_3CHCH_2* + Cl*	1.67E+13	1.17E+09	1.67E+13	1.67E+13
*Pd-CeO_2				
HCl + * → HCl*	1.04E+08	3.11E+13	1.04E+08	2.41E+18
$CH_3CH_2CH_3$ + * → $CH_3CH_2CH_3$*	1.04E+08	1.23E+15	1.04E+08	2.04E+19
CH_3CHCH_2* → CH_3CHCH_2 + *	1.44E+08	1.54E+18	1.44E+08	7.01E+18
HCl* + * → H* + Cl*	1.67E+13	1.64E+13	1.67E+13	1.67E+13
$CH_3CH_2CH_3$* + *→CH_3CHCH_3* + H*	9.42E+08	2.54E+06	1.67E+13	1.67E+13

CH_3CHCH_3* + Cl*→$CH_3CHClCH_3$* + *	1.67E+13	1.21E+04	1.67E+13	1.67E+13
$CH_3CHClCH_3$* + * → $CH_3CHClCH_2$* + H*	9.06E+09	9.06E+09	1.67E+13	1.67E+13
$CH_3CHClCH_2$* + *→CH_3CHCH_2* + Cl*	1.67E+13	3.69E+08	1.67E+13	1.67E+13
*Pt-CeO_2				
HCl + * → HCl*	1.04E+08	3.11E+13	1.04E+08	2.41E+18
$CH_3CH_2CH_3$ + * → $CH_3CH_2CH_3$*	1.04E+08	5.48E+14	1.04E+08	2.04E+19
CH_3CHCH_2* → CH_3CHCH_2 + *	1.44E+08	1.54E+18	1.44E+08	7.01E+18
HCl* + * → H* + Cl*	1.67E+13	1.64E+13	1.67E+13	1.67E+13
$CH_3CH_2CH_3$* + *→CH_3CHCH_3* + H*	3.29E+08	6.57E+05	1.67E+13	1.67E+13
CH_3CHCH_3* + Cl*→$CH_3CHClCH_3$* + *	1.67E+13	4.93E+06	1.67E+13	1.67E+13
$CH_3CHClCH_3$* + * → $CH_3CHClCH_2$* + H*	2.92E+11	8.18E+08	1.67E+13	1.67E+13
$CH_3CHClCH_2$* + *→CH_3CHCH_2* + Cl*	1.67E+13	4.50E+06	1.67E+13	1.67E+13
*Cu-CeO_2				
HCl + * → HCl*	1.04E+08	3.11E+13	1.04E+08	2.41E+18
$CH_3CH_2CH_3$ + * → $CH_3CH_2CH_3$*	1.04E+08	1.06E+15	1.04E+08	2.04E+19
CH_3CHCH_2* → CH_3CHCH_2 + *	1.44E+08	1.54E+18	1.44E+08	7.01E+18
HCl* + * → H* + Cl*	1.67E+13	1.64E+13	1.67E+13	1.67E+13
$CH_3CH_2CH_3$* + *→CH_3CHCH_3* + H*	1.35E+08	6.98E+05	1.67E+13	1.67E+13
CH_3CHCH_3* + Cl*→$CH_3CHClCH_3$* + *	1.67E+13	1.46E+00	1.67E+13	1.67E+13
$CH_3CHClCH_3$* + * → $CH_3CHClCH_2$* + H*	2.88E+11	7.59E+08	1.67E+13	1.67E+13
$CH_3CHClCH_2$* + *→CH_3CHCH_2* + Cl*	1.67E+13	1.09E+02	1.67E+13	1.67E+13

DRC analysis

The degree of rate control (DRC) analysis on all the catalytic surfaces is performed for the contribution of an elementary step to the overall reaction. The summation of DRC coefficient for all elementary steps gives unity; the DRC analysis for elementary steps is given in the table below.

Table S3. Shows the DRC values for elementary steps at 773 K.

**Reactions**	**DRC**								
	**CeO_2**	**V-CeO_2**	**Mn-CeO_2**	**Fe-CeO_2**	**Co-CeO_2**	**Ni-CeO_2**	**Pd-CeO_2**	**Pt-CeO_2**	**Cu-CeO_2**
HCl* + *→ H* + Cl*	-1.47E-02	2.56E-03	6.56E-03	8.51E-04	4.80E-04	4.25E-04	4.06E-04	4.58E-04	9.87E-04
$CH_3CH_2CH_3$* + * → CH_3CHCH_3* + H*	9.19E-01	1.04E+00	1.10E+00	1.01E+00	1.01E+00	1.01E+00	1.01E+00	1.01E+00	1.02E+00
CH_3CHCH_3* + Cl* → $CH_3CHClCH_3$* + *	9.56E-08	6.72E-11	8.10E-11	1.08E-08	1.37E-08	8.11E-09	-3.31E-08	2.73E-08	1.65E-11
$CH_3CHClCH_3$* + *→ $CH_3CHClCH_2$* + H*	5.19E-01	3.28E-08	1.86E-07	8.27E-09	2.44E-09	-3.74E-08	2.81E-08	-1.90E-09	-1.18E-11
$CH_3CHClCH_2$* + *→ CH3CHCH2* + Cl*	7.79E-10	1.97E-11	-1.48E-10	-2.17E-12	-1.56E-09	-7.11E-09	2.37E-08	1.77E-08	-6.69E-12
H* + OH* → H_2O*+ *	1.06E-01	-3.98E-02	-1.01E-01	-1.31E-02	-7.22E-03	-6.32E-03	-6.02E-03	-6.86E-03	-1.52E-02
H* + Cl* → HCl* + *	-1.79E02	3.42E-03	8.75E-03	1.14E-03	6.41E-04	5.68E-04	5.43E-04	6.12E-04	1.32E-03
$\sum_i xi$	0.99	1.0	1.00	1.00	1.00	1.00	1.00	1.00	1.00
